# Diagnosing Dengue at the Point-of-Care: Utility of a Rapid Combined Diagnostic Kit in Singapore

**DOI:** 10.1371/journal.pone.0090037

**Published:** 2014-03-19

**Authors:** Victor C. Gan, Li-Kiang Tan, David C. Lye, Kwoon-Yong Pok, Shi-Qi Mok, Rachel Choon-Rong Chua, Yee-Sin Leo, Lee-Ching Ng

**Affiliations:** 1 Institute of Infectious Disease and Epidemiology, Tan Tock Seng Hospital, Singapore, Singapore; 2 Environmental Health Institute, National Environment Agency, Singapore, Singapore; 3 Yong Loo Lin School of Medicine, National University of Singapore, Singapore, Singapore; 4 School of Biological Sciences, Nanyang Technological University, Singapore, Singapore; Tulane School of Public Health and Tropical Medicine, United States of America

## Abstract

WHO recommendations for dengue diagnosis require laboratory facilities. Antibody-based rapid diagnostic tests (RDTs) have performed poorly, and clinical diagnosis remains the mainstay in dengue-endemic countries. We evaluated a combination antigen-antibody RDT for point-of-care testing in a high-prevalence setting. In this prospective cohort study, adults were enrolled from a tertiary infectious disease centre for evaluation of undifferentiated febrile illness from October 2011 to May 2012. SD Bioline Dengue Duo was evaluated at point-of-care against a WHO-based reference standard of viral isolation, RT-PCR, NS1-, IgM-, and IgG-ELISA. 246 adults were enrolled (median age 34 years, range 18–69), of which 197 could be confirmed definitively as either dengue or non-dengue. DENV-2 was the predominant serotype (79.5%) and the ratio of primary to secondary cases was 1∶1.1. There were no test failures and minimal interobserver variation with a Fleiss’ kappa of 0.983 (95% CI 0.827–1.00). Overall sensitivity and specificity were 93.9% (95% CI 88.8–96.8%) and 92.0% (95% CI 81.2–96.9%) respectively. Using WHO clinical criteria alone for diagnosis had similar sensitivities (95.9%, 95% CI 91.4–98.1%) and lower specificities (20.0%, 95% CI 11.2–33.0%). No significant difference in performance was found when testing early versus late presenters, primary versus secondary cases, or DENV-1 versus DENV-2 infections. The use of a combination RDT fulfills WHO ASSURED criteria for point-of-care testing and can enhance dengue diagnosis in an endemic setting. This has the potential to markedly improve clinical management of dengue in the field.

## Introduction

Dengue is an important vector-borne disease, with over one billion people at risk in the subtropics and tropics [1]. With most of the at-risk population distant from laboratory support, rapid diagnosis at the point-of-care is needed for accurate diagnosis and early clinical intervention. Nucleic acid amplification tests and antigen/antibody detection by enzyme-linked immunosorbent assay (ELISA) represent advances in dengue diagnostics validated in large multicentre trials [2], [3]. However, neither of these modalities is suitable for rapid field diagnosis to triage and determine clinical siting. Thus far, rapid diagnostic tests (RDTs) have performed suboptimally when evaluated in centralised laboratories [4], [5].

Dengue non-structural protein 1 (NS1) antigen testing has proven in multicentre trials to be highly specific although its sensitivity is affected by timing and secondary infection. Thus far, evaluation has been mostly laboratory-based, and studies have been small, with varying reference standards and population cohorts, as reviewed by Blacksell [4]. One recent study assessed the use of point-of-care testing in a high-prevalence paediatric population, showing >90% sensitivity and specificity when performed in a laboratory but lower accuracy when performed on-site in the hospital on the same patients [6].

Diagnostic decision making is often guided by clinical criteria which are not specific [7], [8]. Overlap with other febrile illness especially in the early phase is significant. The validation of a reliable and accessible rapid diagnostic test is critical for managing acute febrile illness in the tropics as well as in returned travelers and with its spread to the developed world, e.g. the United States [9]. While empirical management for dengue illness is feasible in the absence of confirmation, a positive dengue test with a compatible clinical syndrome would reduce the urgency for testing or empirical treatment for other tropical fevers such as typhoid or leptospirosis and may guide hospitalization requirements.

In search of a rapid diagnostic tool to be used at the point-of-care, we performed an evaluation of combination rapid testing combining NS1 antigen and IgM/IgG detection against a WHO-based composite reference standard based on serial blood sampling. SD Dengue Duo (Standard Diagnostics, Inc., Gyeonggi-do, Korea) was chosen, as it has shown good performance in multiple laboratory-based trials with sensitivities and specificities ranging from 75.5–92.9% and 88.8–100% respectively using frozen serum or plasma samples [10]–[14]. We evaluated this commercially available assay using whole blood to obtain a reading within 30 minutes of patient presentation.

## Methods

We followed evaluation criteria by the World Health Organisation and Special Programme for Training and Research in Tropical Diseases (WHO/TDR) Diagnostics Evaluation Expert Panel [15], [16], and Standards for the Reporting of Diagnostic accuracy studies (STARD) guidelines [17].

### Ethics Statement

Ethical approval was provided by the Domain Specific Review Board of the National Healthcare Group, Singapore (DSRB/E/2009/432) and written informed consent obtained from all subjects.

### Study Design

We enrolled adult patients who consented to take part in the Prospective Adult Dengue Study, a cohort study of acutely febrile adults at the Communicable Disease Centre, Tan Tock Seng Hospital, Singapore from October 2011 to May 2012. These comprised referrals for fever for investigation from the emergency department, other medical institutions, or self referrals to the Communicable Disease Centre. Inclusion criteria were age 18 years and above with an acute undifferentiated febrile illness (recorded temperature >37.5°C with no alternative syndromic diagnosis determined by treating clinician). Pregnant women were excluded from the study as obstetric cases are routinely managed at a different hospital. Point-of-care testing was done on-site at phlebotomy using whole blood on first presentation. Concurrently, plasma samples taken daily during acute illness were sent to the Environment Health Institute, Singapore for diagnostic testing. Subjects were also assessed for convalescent serology 21 to 30 days after their initial presentation. Detailed demographic, clinical, and laboratory data were prospectively collected following our research schedule.

### Laboratory Testing

All confirmatory testing was performed at the Environment Health Institute, Singapore, a WHO Collaborating Centre for Reference and Research on Arbovirus and its Associated Vectors. The laboratory was blinded to the results of the point-of-care test. Plasma samples were subject to a two-stage real-time reverse transcriptase polymerase chain reaction comprising screening using SYBR green followed by a tetraplex probe-based serotype detection assay [18]. The serological suite used was: Platelia™ NS1 ELISA (Bio-Rad Laboratories, Marnes-la-Coquette, France), Panbio® Dengue IgG Indirect, IgG Capture, and IgM Capture ELISAs (Alere Inc., Waltham, MA, USA). Viral isolation in C6/36 cells and confirmatory immunofluorescence testing for serotype identification were performed for all samples as previously described [18].

Classification of final dengue status was according to the following WHO-based composite standard [19], [20]. Definite exclusion of acute dengue was made for subjects negative by PCR, NS1, and IgM, but may be IgG Indirect positive without four-fold rise in titre by convalescent visit at 21–30 days. Non-conclusive cases were defined as both PCR and NS1 negative, with either IgM or IgG Capture testing positive in the acute phase and no evidence of seroconversion. These were classified as reference test indeterminate and not used in evaluating the sensitivity and specificity of the assay. Definite confirmation of current DENV infection was made when testing by PCR or NS1 was positive, or with IgM or IgG Indirect seroconversion, or with four-fold rise in IgG Indirect by titration, or were positive by viral isolation.

Confirmed cases with samples that are negative by IgG Indirect or Capture assay during the acute phase were classified as primary cases. An acute sample positive by IgG Capture or an early acute (on or before day 5 of illness) sample positive by IgG Indirect defined secondary infection status in confirmed dengue cases. Cases that could not be classified, such as those missing convalescent samples, were labeled primary/secondary status indeterminate and were not used in evaluating the point-of-care test (POCT) for performance in primary compared with secondary dengue.

### Point-of-care Evaluation

SD Dengue Duo is a commercially available lateral-flow diagnostic assay with separate cassettes for NS1 detection and IgM/IgG detection. Kits were provided by Standard Diagnostics, Inc. (Gyeonggi-do, Korea) and stored at room temperature (20–34°C). Tests were performed according to the manufacturer’s instructions at routine phlebotomy by research assistants using whole blood from venepucture at the clinic where the patient was managed clinically. Research assistants were trained by laboratory personnel from the Environment Health Institute in a single training session lasting 2 hours prior to study initiation. Briefly, after venepuncture, whole blood was transferred to the test cassettes using provided disposable micropipettes and the test was read after 20 minutes. A log book was maintained of problems encountered during testing. In the initial phase of the study (October 2011 to February 2012), each strip was read by two independent blinded readers to determine inter-rater reliability. Discrepancy between the two readers was resolved by the reading of an independent blinded third reader, whose decision was final. Readers did not have access to any diagnostic information from the external reference laboratory at time of reading. Case definitions for probable dengue by clinical criteria used were WHO 1997 (fever with any two of headache, retro-orbital pain, myalgia, arthralgia, rash, haemorrhagic manifestations, or leukopaenia) [21] and WHO 2009 (fever with two of nausea or vomiting; rash; aches and pains; tourniquet test positive; leucopenia; any warning sign) [19].

### Sample Size Calculation

Sample size was calculated using an estimated prevalence of dengue positive cases at 60% based on routinely collected information. Sensitivity analysis for sample size calculation was performed using the published 95% confidence intervals for sensitivity and specificity in the two studies that analysed the combined use of NS1/IgM/IgG components of the SD Dengue Duo in the laboratory setting [10], [13]. For 95% confidence and 10% precision, the minimum estimate of sample size required was 151.

### Statistical Analysis

Inter-rater agreement between blinded readers of each test strip in the initial phase was calculated using Fleiss’ kappa for multi-rater agreement when a fixed number of raters is drawn from a large pool of raters [22]. Confidence intervals for sensitivities and specificities were calculated according to the Wilson score method without continuity correction [23]. For correlated proportions when comparing different tests done on the same cohort, McNemar’s test was used for hypothesis testing [24], and Tango’s score confidence interval for a difference of proportions with matched pairs was determined [25]. For comparing sensitivities and specificities in different subgroups, the Newcombe-Wilson method without continuity correction was used to construct a confidence interval for difference in proportion [26], and hypothesis testing was performed using Fisher’s exact test. Level of significance was set at 5% for all hypothesis testing.

## Results

### Demographics

**Figure 1:**
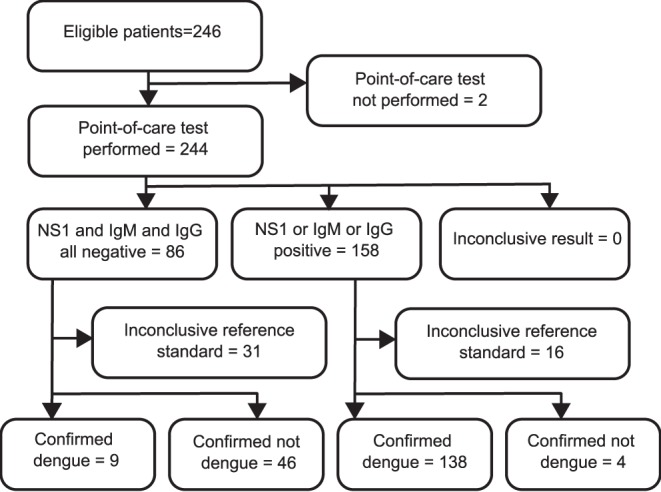
Flow diagram of recruited subjects in the evaluation of point-of-care testing against a laboratory-based composite reference standard.

From October 6, 2011 to May 31, 2012, 246 consecutive patients were enrolled. Of these 246 subjects, 2 did not have point-of-care testing performed for logistical reasons and were excluded from subsequent analysis (Figure 1).

Definite confirmation of current DENV infection as previously defined was obtained in 147 (60.2%), definite exclusion of acute dengue in 50 (20.2%), and in 47 (19.2%) dengue diagnostics were non-conclusive. Of the latter, 26 (10.7%) had serological evidence of recent DENV infection without four-fold rise in titres or virus detection, and 21 (8.6%) were negative by virus detection and acute serology without convalescent visit to assess rise in antibody titres. In the 50 where dengue had been definitively excluded, further workup at the discretion of the treating clinicians found confirmed influenza in 3 and rickettsiosis in 4 cases; in the remainder, an infective aetiology was not identified. Of the confirmed dengue cases, 22 were serotyped as DENV-1, 89 as DENV-2 (79.5%), and one as DENV-3, reflecting the DENV-2 predominance in Singapore since 2007 [27]. The ratio of primary to secondary cases was 1∶1.1.

The median age for the study cohort was 34 years (range 18–69 years). The majority (78.3%) was male as previously described in Singapore [28] and the Western Pacific [29]. The majority (83.8%) of subjects were tested in ambulatory care with the remainder admitted under the infectious disease service from the emergency department. The median time from fever onset to presentation was 6 days (range 1–14 days) with 30.5% presenting within the first 5 days of illness. Among the 147 confirmed dengue patients, 119 had dengue fever and 28 had dengue haemorrhagic fever by WHO 1997 criteria; 70 had probable dengue without warning signs, 77 probable dengue with warning signs, and five had severe dengue by WHO 2009 criteria. As modified WHO 1997 criteria remain in use in the South East Asia region [30] and describe a distinct pathophysiological syndrome compared with the broader remit of the WHO 2009 classification, we retain descriptive analysis using both criteria.

### Interobserver Variation in Reading SD Dengue Duo Point-of-care Test

Inter-rater agreement between two blinded readers reporting on the visual result from the same test kit was analysed using 158 tests performed in the initial phase as described above. All tests were completed according to the manufacturer’s instructions with no difficulties recorded, with adequate sample migration and appearance of the immunochromatographic control in 100% of tests. A positive result was determined in three ways: (i) any of NS1/IgM/IgG detected; (ii) either NS1/IgM detected, and; (iii) NS1 detected only. Inter-rater agreement was assessed for each of these. Using (i), there was one disagreement between readers in test interpretation (Fleiss’ kappa = 98.3%, 95% CI 82.7–100%). With (ii), there was also a single disagreement giving rise to the same Fleiss’ kappa statistic. If only the NS1 component was used to determine a positive test as in (iii), there were no disagreements, giving a kappa = 1.00 (95% CI 84.4–100%).

### Performance of SD Dengue Duo Point-of-care Test Compared to Reference Standard

The composite reference standard described above was used to classify cases into definite confirmation of current DENV infection and definite exclusion of acute dengue. The performance of the SD Dengue Duo kit was compared against this reference standard, giving a sensitivity of 93.9% and specificity of 92.0% when used as designed to determine acute DENV infection by a positive reading in any one of the NS1, IgM, or IgG components performed in the clinic or at bedside (Table 1).

**Table 1:** Performance of point-of-care strategies for dengue diagnosis against laboratory-based composite reference standards.

	Sensitivity	Specificity	Positive predictivevalue	Negative predictivevalue
**Dengue Duo (NS1/IgM/IgG)**	138/147, 93.9 (88.8–96.8)	46/50, 92.0 (81.2–96.9)	138/142, 97.2 (93.0–98.9)	46/55, 83.6 (71.7–91.1)
**Dengue Duo (NS1/IgM)**	135/147, 91.8 (86.3–95.3)	48/50, 96.0 (86.5–98.9)	135/137, 98.5 (94.8–99.6)	48/60, 80.0 (68.2–88.2)
**Dengue Duo (NS1 only)**	120/147, 81.6 (74.6–87.1)	49/50, 98.0 (89.5–99.7)	120/121, 99.2 (95.5–99.9)	49/76, 64.5 (53.3–74.3)
**WHO 1997**	141/147, 95.9 (91.4–98.1)	10/50, 20.0 (11.2–33.0)	141/181, 77.9 (71.3–83.3)	10/16, 62.5 (38.6–81.5)
**WHO 2009**	142/147, 96.6 (92.3–98.5)	13/50, 26.0 (15.9–39.6)	142/179, 79.3 (72.8–84.6)	13/18, 72.2 (49.1–87.5)
**WHO 1997 then Dengue Duo (NS1/IgM/IgG)**	134/147, 91.2 (85.5–94.8)	47/50, 94.0 (83.8–97.9)	134/137, 97.8 (93.8–99.3)	47/60, 78.3 (66.4–86.9)
**WHO 2009 then Dengue Duo (NS1/IgM/IgG)**	134/147, 91.2 (85.5–94.8)	47/50, 94.0 (83.8–97.9)	134/137, 97.8 (93.8–99.3)	47/60, 78.3 (66.4–86.9)

Data are the number correct/number tested, % (95% confidence interval).

There was no significant difference between the sensitivities using any of the three components compared with using NS1 OR IgM (p = 0.25), but there was a significant drop in sensitivity by 12.3% (95% CI 7.9–18.5%) when using only NS1 (p<0.001). There was no significant difference in the specificities (p> = 0.25).

We also compared SD Dengue Duo with clinical criteria for dengue diagnosis recommended by the WHO in 1997 and 2009 [19], [21]. A total of 181 (91.9%) and 179 (90.9%) of the study population satisfied the clinical definition of dengue fever from WHO 1997 and 2009 respectively, with sensitivities and specificities in Table 1 when measured against the reference panel. Comparing the sensitivities against those of the point-of-care test with any of NS1, IgM, or IgG indicating a positive result, the sensitivities of the clinical criteria were not statistically different (p = 0.549 and p = 0.388 for WHO 1997 and 2009 respectively), while the specificities were significantly better with the point-of-care test (p<0.001 for both) with a difference in specificity of 72.0% (95% CI 54.9–83.0%) using WHO 1997 criteria and 66% (95% CI 49.1–78.1%) using WHO 2009 criteria. Using the NS1 component alone, while significantly more specific than clinical criteria (p<0.001), it was also significantly less sensitive by 14.3% (95% CI 8.5% to 21.3%; p<0.001) compared to WHO 1997 criteria.

Finally we analysed the use of the POCT in combination with WHO clinical criteria by using either WHO 1997 or WHO 2009 probable dengue criteria as the initial screening method followed by Dengue Duo (NS1/IgM/IgG) for those who were positive by the WHO criteria. This two step strategy showed no significant difference in performance from using the POCT alone and significant increase in specificity compared to using clinical criteria alone.

To analyse the performance of the test in early versus late presentations, time from fever onset was dichotomised to less than or equal to 5 days and greater than 5 days. The sensitivity of SD Dengue Duo using any of the three testing strategies was not significantly lower in early presenters (Table 2). The difference in specificities (early presenters 100%, 95% CI 72.3%–100%; late presenters 90.0%, 95% CI 77.0%–96.0%) was similarly non significant (p = 0.571). Comparing the two main dengue serotypes, Dengue Duo did not perform differently in DENV-1 compared with DENV-2 (p>0.5 for difference in sensitivity using all three testing strategies).

**Table 2:** Sensitivity of SD Dengue Duo in different subpopulations against laboratory-based composite reference standards.

	POCT NS1 OR IgM OR IgG	POCT NS1 OR IgM	POCT NS1
**Fever < = 5 days (n = 50)**	45/50, 90.0 (78.6–95.7)	44/50, 88.0 (76.2–94.4)	43/50, 86.0 (73.8–93.1)
**Fever >5 days (n = 97)**	93/97, 95.9 (89.9–98.4)	91/97, 93.8 (87.2–97.1)	77/97, 79.4 (70.3–86.2)
**DENV-1 (n = 22)**	22/22, 100.0 (85.1–100.0)	21/22, 95.5 (78.2–99.2)	19/22, 86.4 (66.7–95.3)
**DENV-2 (n = 89)**	84/89, 94.4 (87.5–97.6)	84/89, 94.4 (87.5–97.6)	78/89, 87.6 (79.2–93.0)

Data are the number correct/number tested, % (95% confidence interval).

We also compared the sensitivities of the different POCT strategies in primary (n = 62) and secondary (n = 68) confirmed dengue cases (data not shown). While there was a trend to a lower sensitivity in secondary cases, the difference was not statistically significant using any of the POCT strategies (p>0.5 in all cases).

## Discussion

Dengue is in need of improved diagnostics. The current WHO recommendations for laboratory diagnosis are viral isolation, RT-PCR, or NS1 detection in early illness, and a four-fold rise of dengue-specific antibody titres for patients presenting later [19]. NS1 antigen detection has been intensively evaluated in the ELISA and rapid test platforms in the last decade, and recommended as a confirmatory test in recent expert reviews [1], [20], [31]. However, these laboratory-based assays do not address the clinical requirement that dengue diagnosis needs to be rapid and at the point-of-care given that shock can set in within hours.

Clear differentiation from other acute undifferentiated tropical febrile illness is necessary for appropriate triage to allocate patients to primary, secondary or tertiary care [1]. In a published series from our centre in Singapore, alternative aetiologies for acute fever included malaria, typhus, mumps, enteric fever, measles, rubella, and viral hepatitis [32]. Diagnostic testing done at hospital laboratories using PCR, ELISA, or rapid assays incurs practical hurdles of cost, batch testing, and turn-around time. Clinicians with an immediate need for decision making for patient management may have to rely on clinical diagnostic criteria. Current WHO clinical diagnostic criteria for probable dengue are not specific. In a recent prospective study from Singapore in primary care, the sensitivity of WHO 1997 criteria for dengue fever ranged from 95.0–98.3% and WHO 2009 criteria for probable dengue from 95.9–100.0% for patients 18–55 years old. However, specificity for WHO 1997 ranged from 26.3–35.2% and WHO 2009 from 19.0–23.0%, varying with age [8]. The high sensitivities in both WHO 1997 and 2009 criteria need to be interpreted in the context of potential referral bias for suspected dengue. In a vaccine trial site with comprehensive fever surveillance in Thailand, the sensitivity of the WHO clinical diagnostic criteria for probable dengue was only 46% [33].

WHO first articulated criteria for rapid diagnostic testing in 2003 [34], coining the acronym ASSURED to stand for Affordable, Sensitive, Specific, User-friendly, Rapid & Robust, Equipment-free, and Delivered. Dengue NS1 antigen and antibody combination rapid testing may fulfill these requirements. The paper-based cassette format for immunochromatographic tests costs less than laboratory-based methods of dengue diagnosis. We documented sensitivity and specificity of 93.9% and 92.0% with the lower bound of the 95% confidence interval at 88.8% and 81.2% respectively using a testing strategy where the detection of any of NS1, IgM, or IgG leads to a positive result. The test is user friendly as it can be performed without extensive training using blood derived from a minimally invasive finger prick or venepuncture to obtain a full blood count. The entire procedure takes less than half an hour. We demonstrated robustness with no detected test failures and high inter-observer reliability. No equipment is required other than a lancet or needle. Finally, the test is commercially available with the potential to be successfully delivered to the point-of-care.

Singapore is hyperendemic for dengue with all four serotypes co-circulating, and with mainly young adults presenting with dengue; in 2011, only 7.8% of reported dengue cases in Singapore were below 15 years [35]. This single site study focussed on adults at the main national referral centre as paediatric cases were few and treated at other hospitals. A recent study that evaluated combination RDT at point-of-care in Cambodian children while demonstrating a high positive predictive value, differed from ours in counting probable dengue cases with non-conclusive serological evidence for acute infection as gold-standard positive cases, introducing a possible source of bias [6].

This evaluation assessed the performance of SD Dengue Duo in a high prevalence setting of a tertiary referral centre for febrile illness in a country with high healthcare utilization in a non-outbreak year. The combined prevalence of confirmed and probable dengue of 70.5% in the study cohort will not be reflective of the situation in primary care where the prior probability of dengue will be lower, but may be similar to that in an outbreak. The combination of antigen and antibody detection has allowed for comparable performance of the kit both early and late in illness, as previously demonstrated [14], [36]. Concerns that NS1 detection may be poorer in secondary infection [37] were not borne out using any of the testing strategies in the POCT approach in our population. While combination with antibody testing may have ameliorated the poor performance of NS1 detection in secondary cases, another potential source of bias in our study compared to previous reports was the preponderance of probable dengue cases excluded from analysis as reference test indeterminate which were secondary cases (20 of 26). These were mainly late presenters without detectable virus and without significant change between acute and convalescent antibody titre. Our exclusion of such indeterminate cases from our analysis while likely biasing the sensitivity and specificity estimates upwards ameliorates the uncertainty around whether indeterminate cases represent acute or only recent infection. Modeling approaches may be required to take the impact of these and other sources of diagnostic bias into account and provide more accurate estimates of test performance [38]. The recent Cambodian evaluation demonstrates that high antibody levels by haemagglutination inhibition assay reduce the sensitivity of NS1 detection, counting serologically probable cases as positive. We do not find that IgG positivity on the day of testing significantly affects the sensitivity of NS1 detection in our cohort (data not shown); this difference is likely due to the difference in definition of positive cases as discussed above. We were unable to prove equivalent performance between dengue serotypes as the small numbers of DENV-1, DENV-3, and DENV-4 cases did not lead to statistical significance; this is a limitation of the dengue serotype distribution in Singapore and is due to the single-site field trial design. In our setting, this point-of-care testing approach was superior to the use of WHO clinical diagnostic criteria alone for diagnosis and a two-step strategy using clinical criteria to screen did not compromise diagnostic performance. A comprehensive field economic evaluation of such a strategy ideally with a cluster randomized design with and without POCT use in a variety of settings would be required to confirm its superior utility as this study was not planned as an evaluation of its impact on treatment decisions.

In conclusion, this evaluation supports the use of a combination rapid diagnostic test to diagnose dengue in the setting of high disease incidence. In accordance with WHO and STARD guidelines for diagnostic evaluation, we have shown the feasibility and utility of using whole blood sampling on a commercially available kit for true point-of-care diagnosis of dengue. This has the potential to improve clinical management of dengue as well as reduce its global morbidity and mortality.
